# Supplementary figures and images for: Molecular Interaction of Genes Related to Anthocyanin, Lipid and Wax Biosynthesis in Apple Red-Fleshed Fruits
Source: Int J Mol Sci. 2025 Nov 13;26(22):10987. doi: 10.3390/ijms262210987 (PMC12652052; doi:10.3390/ijms262210987)

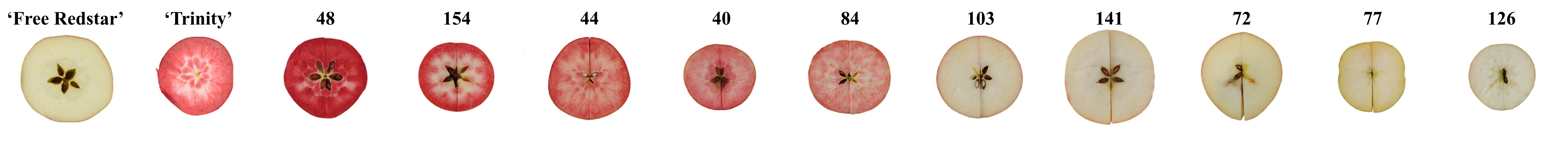

Supplement: Supplementary file 1 [file ijms-26-10987-s001.zip › Fig_ S12_fruit flesh coclor visualization.jpg]

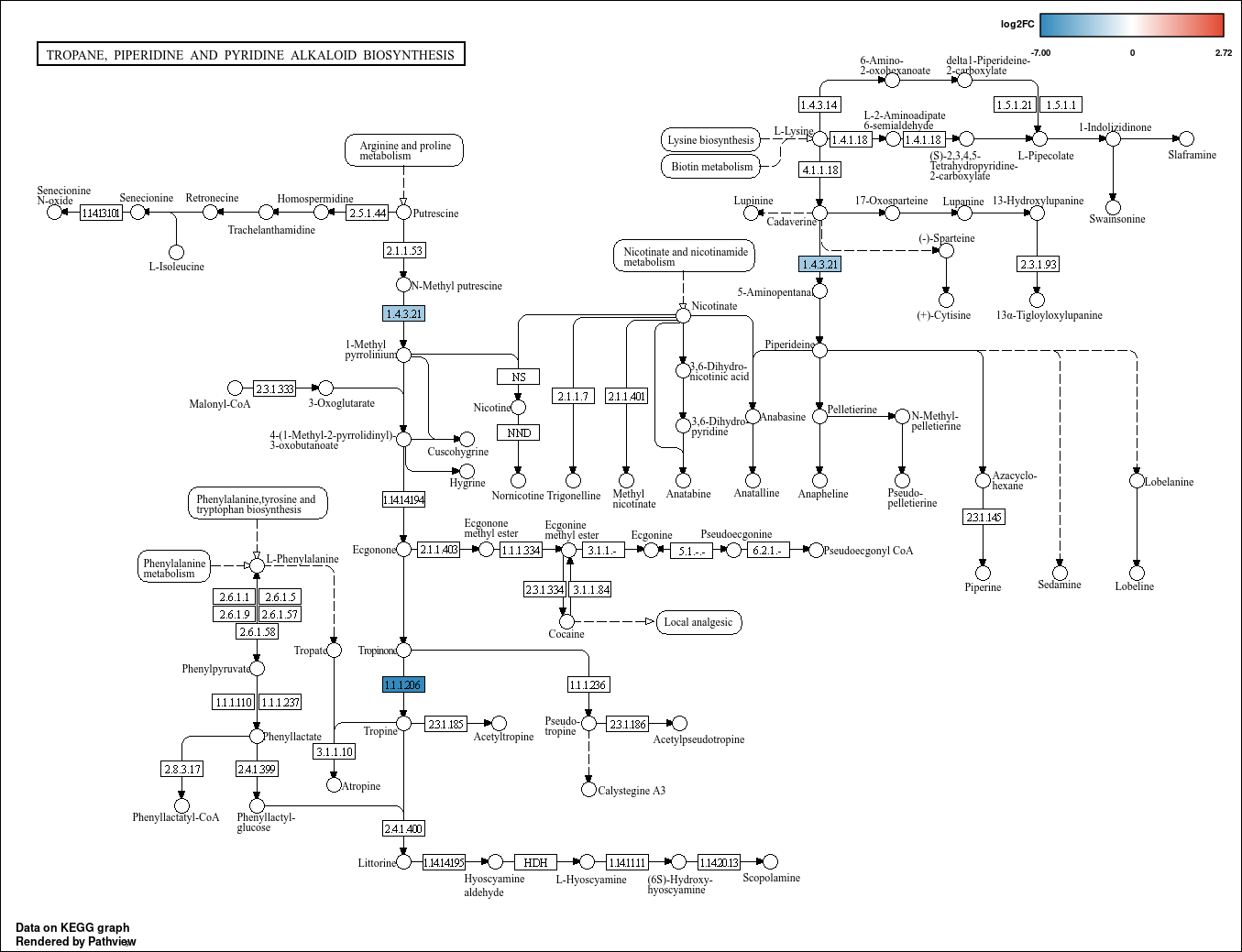

Supplement: Supplementary file 1 [file ijms-26-10987-s001.zip › Fig_S10_mdm00960.02_DESeq2_ORA_mdm_v5_norm_realscale.png]

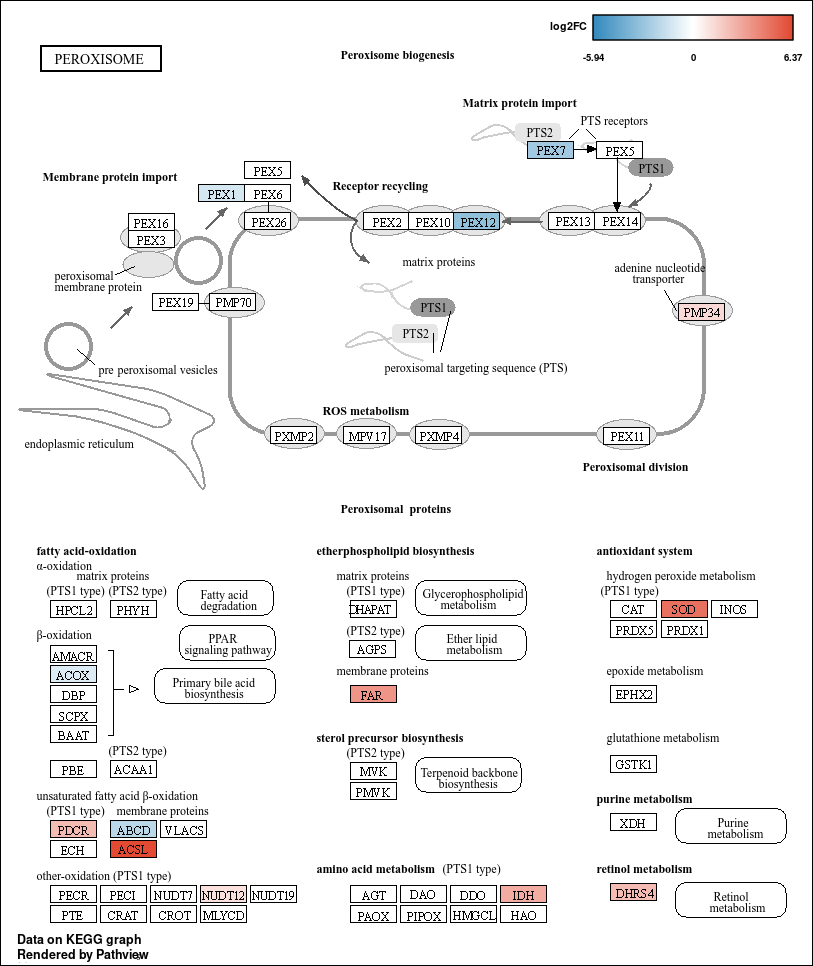

Supplement: Supplementary file 1 [file ijms-26-10987-s001.zip › Fig_S11_mdm04146.02_DESeq2_ORA_mdm_v5_norm_realscale.png]

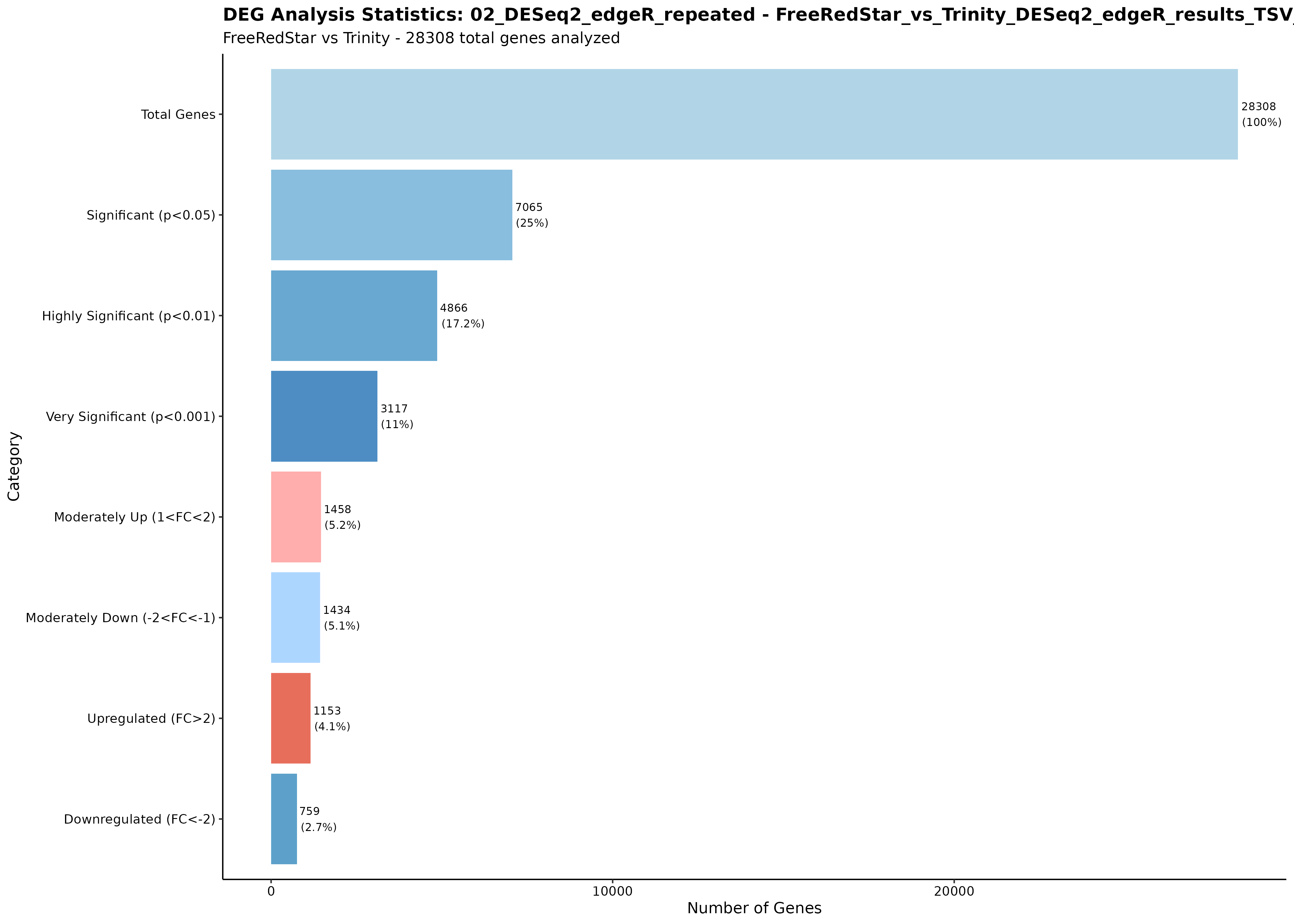

Supplement: Supplementary file 1 [file ijms-26-10987-s001.zip › Fig_S1_01_02_DESeq2_edgeR_repeated - FreeRedStar_vs_Trinity_DESeq2_edgeR_results_TSV_annot__statistics_summary.png]

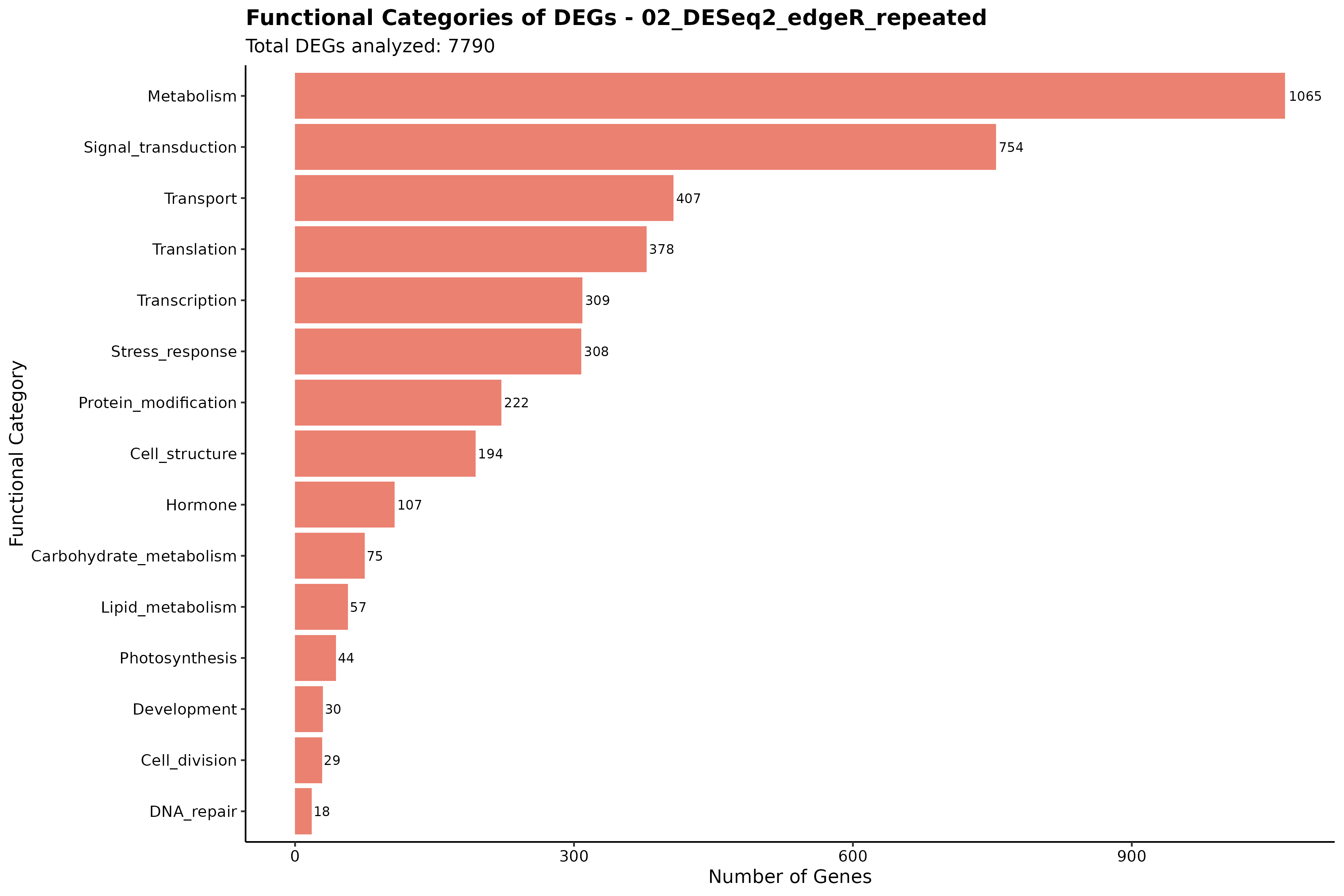

Supplement: Supplementary file 1 [file ijms-26-10987-s001.zip › Fig_S2_functional_categories_barplot.png]

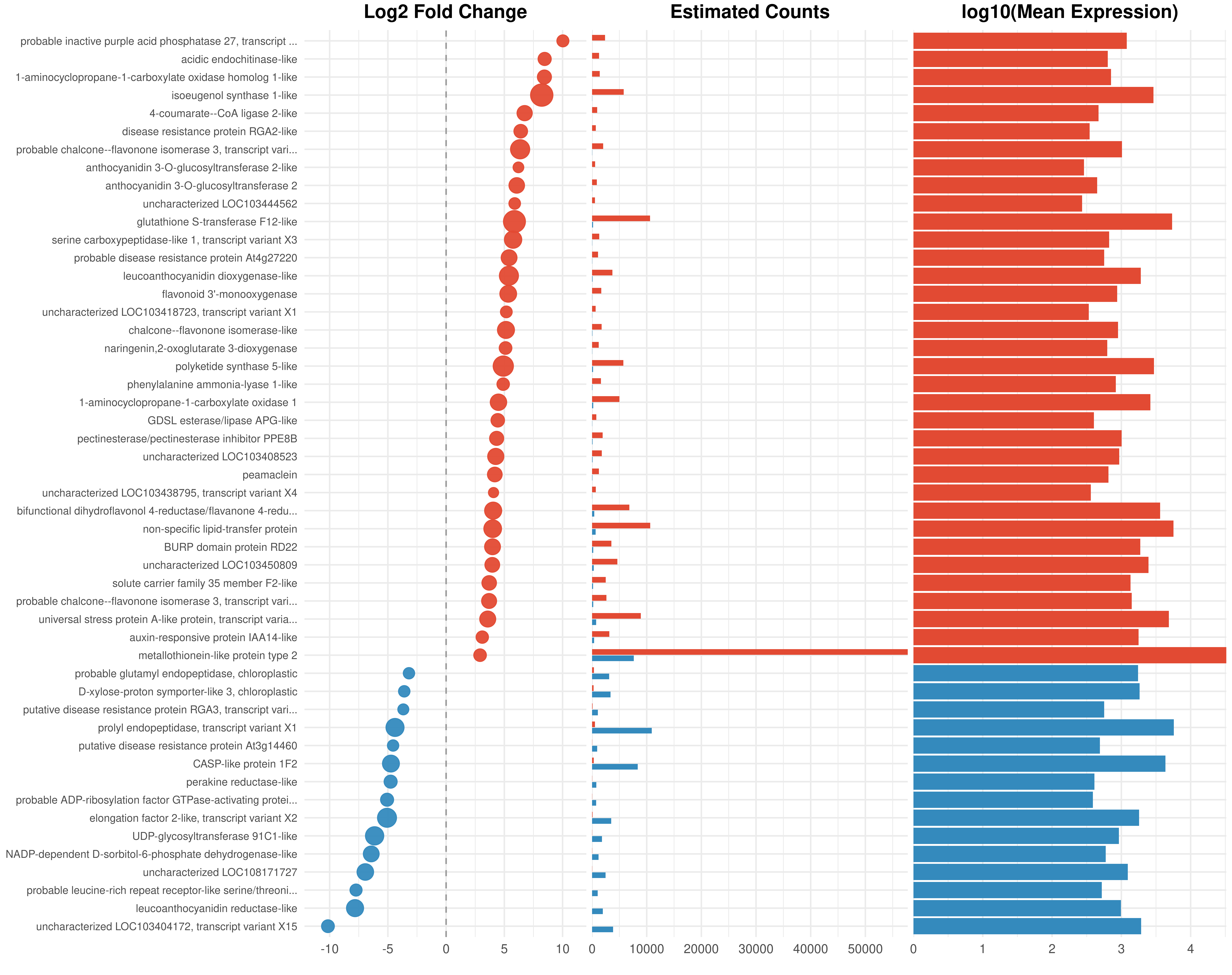

Supplement: Supplementary file 1 [file ijms-26-10987-s001.zip › Fig_S3_02_DESeq2_edgeR_repeated - FreeRedStar_vs_Trinity_DESeq2_edgeR_results_TSV_annot__description_top50_deg_summary.png]

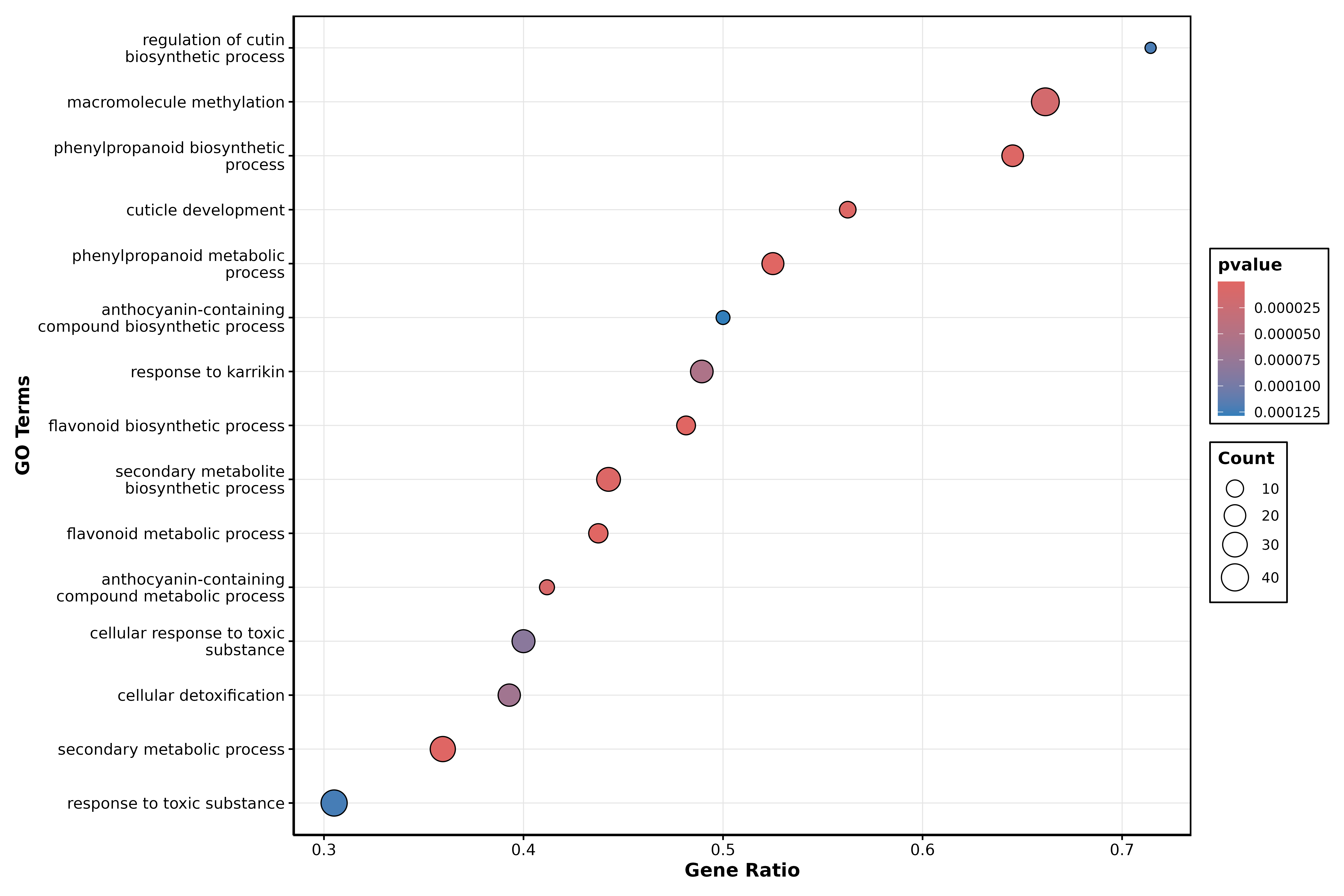

Supplement: Supplementary file 1 [file ijms-26-10987-s001.zip › Fig_S4_go_dotplot_gsea_BP.tiff]

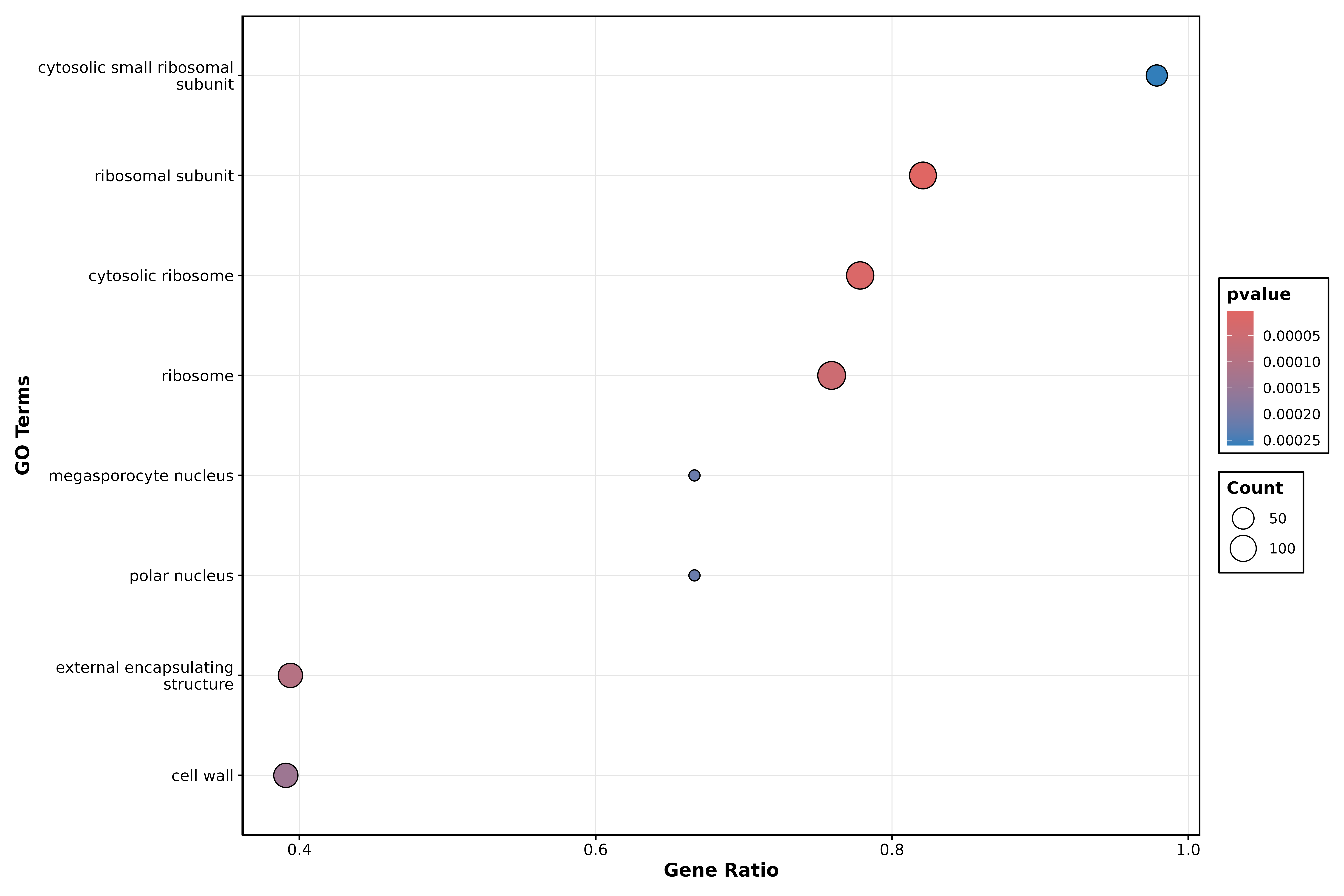

Supplement: Supplementary file 1 [file ijms-26-10987-s001.zip › Fig_S5_go_dotplot_gsea_CC.tiff]

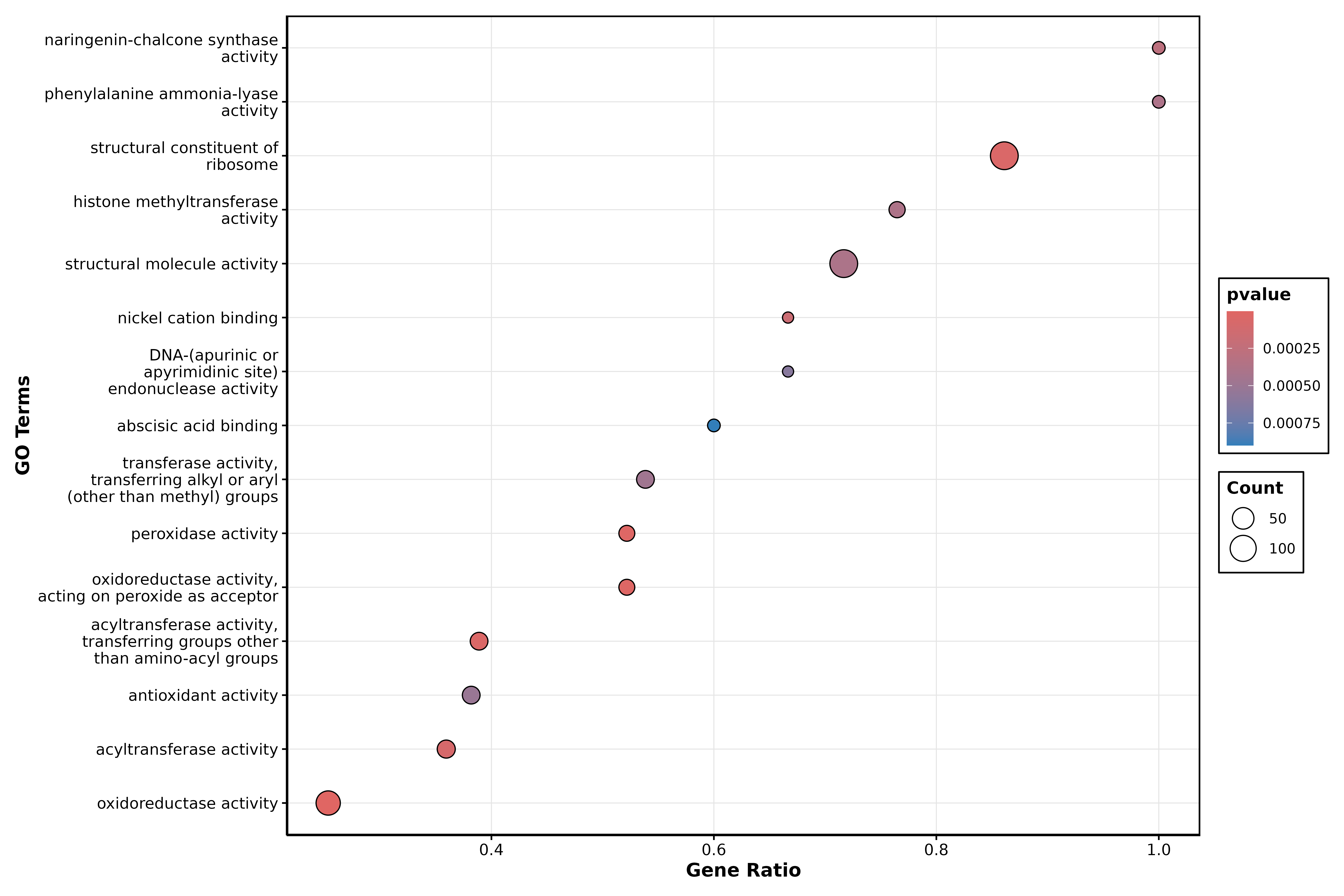

Supplement: Supplementary file 1 [file ijms-26-10987-s001.zip › Fig_S6_go_dotplot_gsea_MF.tiff]

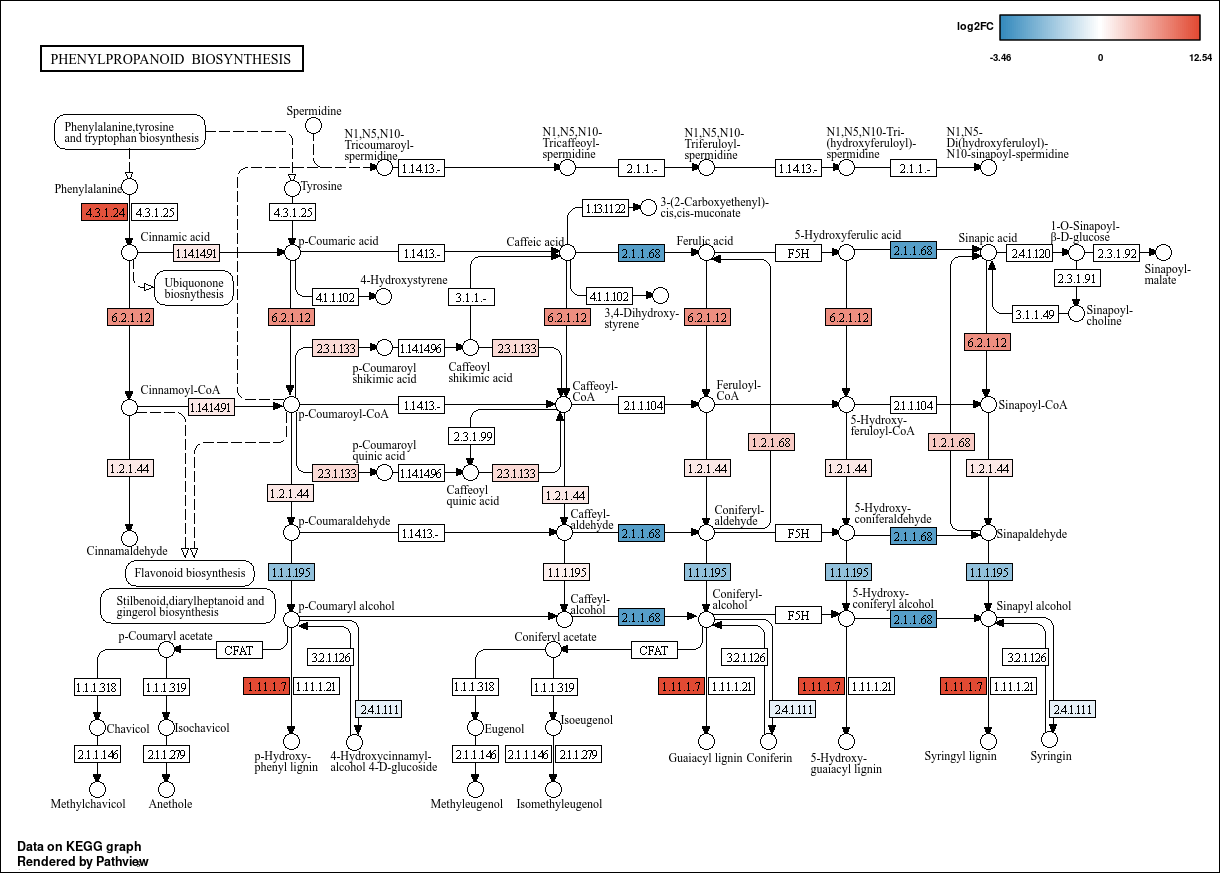

Supplement: Supplementary file 1 [file ijms-26-10987-s001.zip › Fig_S7_mdm00940.02_DESeq2_ORA_mdm_v5_norm_realscale.png]

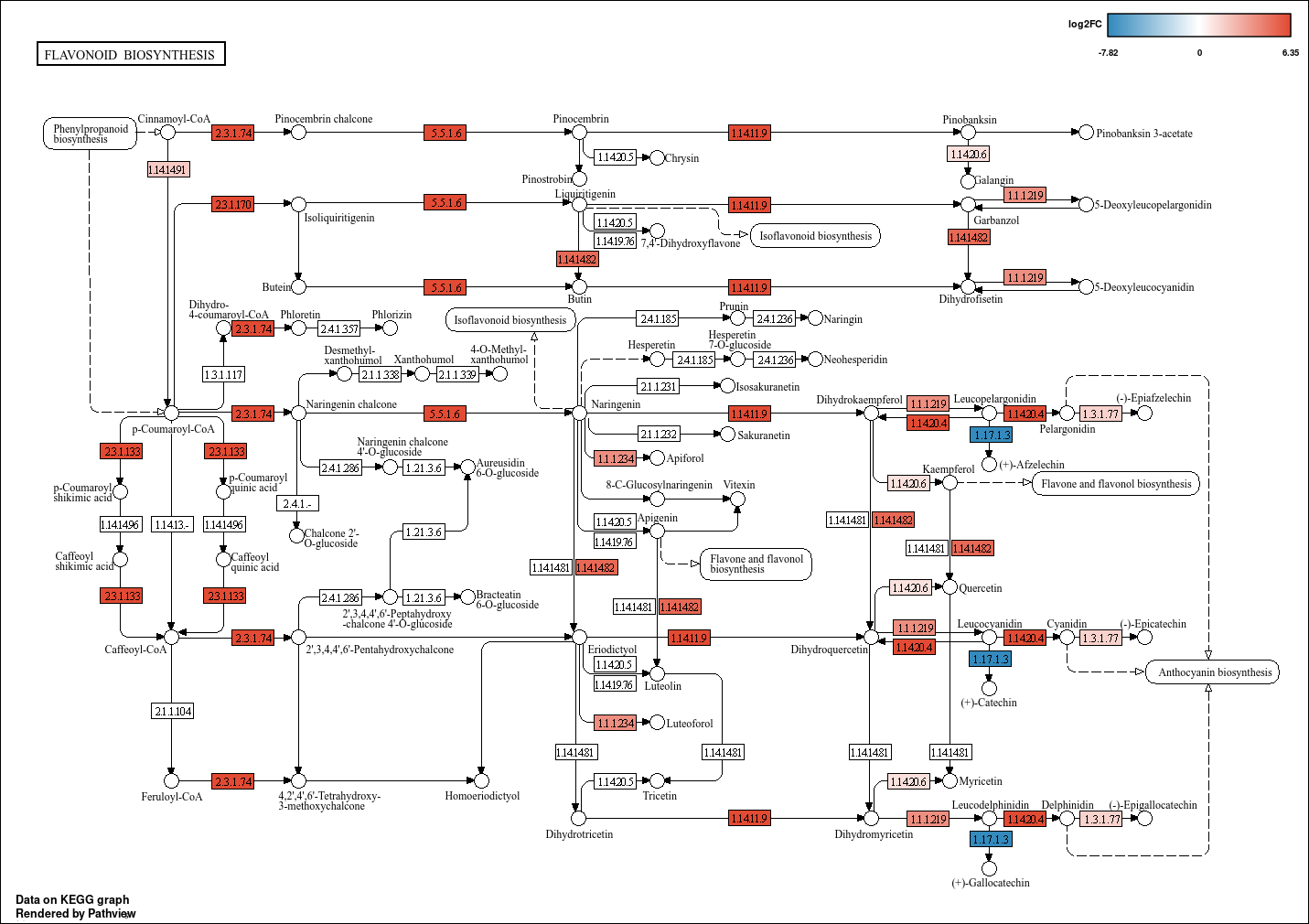

Supplement: Supplementary file 1 [file ijms-26-10987-s001.zip › Fig_S8_mdm00941.02_DESeq2_ORA_mdm_v5_norm_realscale.png]

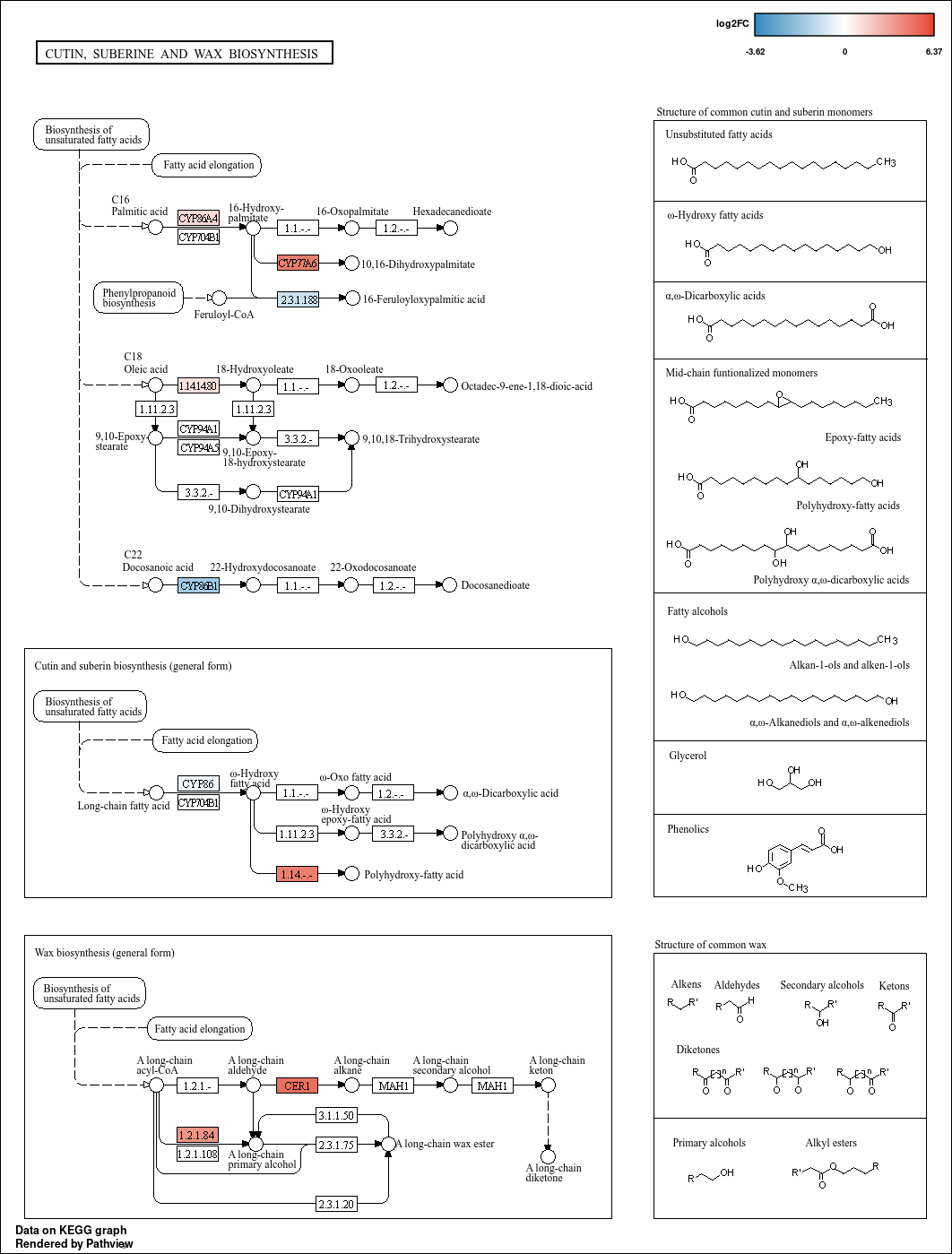

Supplement: Supplementary file 1 [file ijms-26-10987-s001.zip › Fig_S9_mdm00073.02_DESeq2_ORA_mdm_v5_norm_realscale.png]
